# The complete chloroplast genome sequence of leibnitzia anandria (linnaeus) turczaninow

**DOI:** 10.1080/23802359.2024.2347511

**Published:** 2024-05-02

**Authors:** Binghua Ru, Ting Wang, Yongfeng Liu, Xiaochao Zhao, Ming Lei

**Affiliations:** a Shanghai Origingene Biotechnology Company Limited, Shang Hai, China; b GeneMind Biosciences Company Limited, Shen Zhen, China

**Keywords:** Chloroplast genomics, GeneLab M sequencing, medicinal plants, phylogenetic analysis

## Abstract

Leibnitzia anandria is a perennial herbaceous plant with medicinal properties, and the entire plant can be used in traditional medicine. Leibnitzia anandria was once classified under the genus Gerbera Cass., but was reclassified under Leibnitzia Cass. recently. In this study, using the GeneLab M sequencing technology of the Genemind platform, we have sequenced, assembled, and analyzed the complete chloroplast genome of Leibnitzia anandria for the first time. The genome is 154168 bp in length, consisting of a large single-copy region(LSC, 80166 bp), a small single-copy region(SSC, 18202 bp), and a pair of inverted repeat sequences(IR, 27900 bp). We have predicted and annotated a total of 133 genes, including 88 protein-coding genes, 37 tRNA-coding genes, and 8 rRNA-coding genes. The results of the phylogenetic analysis indicate that Leibnitzia anandria and Leibnitzia nepalensis, as well as the closely related Gerbera plant, clustered into a separate clade, rather than grouping together with the other plants belonging to the tribe Mutisieae. This study provides new information for the phylogeny research of Leibnitzia anandria, contributing to a better understanding of its taxonomy and evolution.

## Introduction

Leibnitzia anandria (Linnaeus) Turczaninow (Gerbera anandria _ Linnaeus 1758) is a perennial herbaceous plant and the model species of genus Leibnitzia, belonging to the tribe Mutisieae in the subfamily Carduoideae of the family Asteraceae (Wu and Peng 2004). As a traditional medicinal plant, the whole plant of Leibnitzia anandria can be used as medicine and contains components such as coumarin, with functions of clearing heat and detoxifying, diuresis and reducing swelling, as well as relieving cough and arresting bleeding (Gu et al. 1987, Qiu and Du 2005). Leibnitzia anandria was once classified as Gerbera Cass. (Cheng 1996), but later separated into the independent genus of Leibnitzia Cass. The main difference between Leibnitzia and Gerbera L. lies in the former having two reproductive periods in a year and there are also differences in the inflorescence morphology, leaves and plant size (Wu and Peng 2002). In this study, we sequenced and assembled the chloroplast genome of Leibnitzia anandria for the first time using Genemind platform’s GeneLab M sequencing technology. Through comprehensive annotation and evolutionary analysis, this study provides a reference for understanding its classification and further study.

## Materials and methords

### Plant materials and DNA extraction

**Figure 1.**
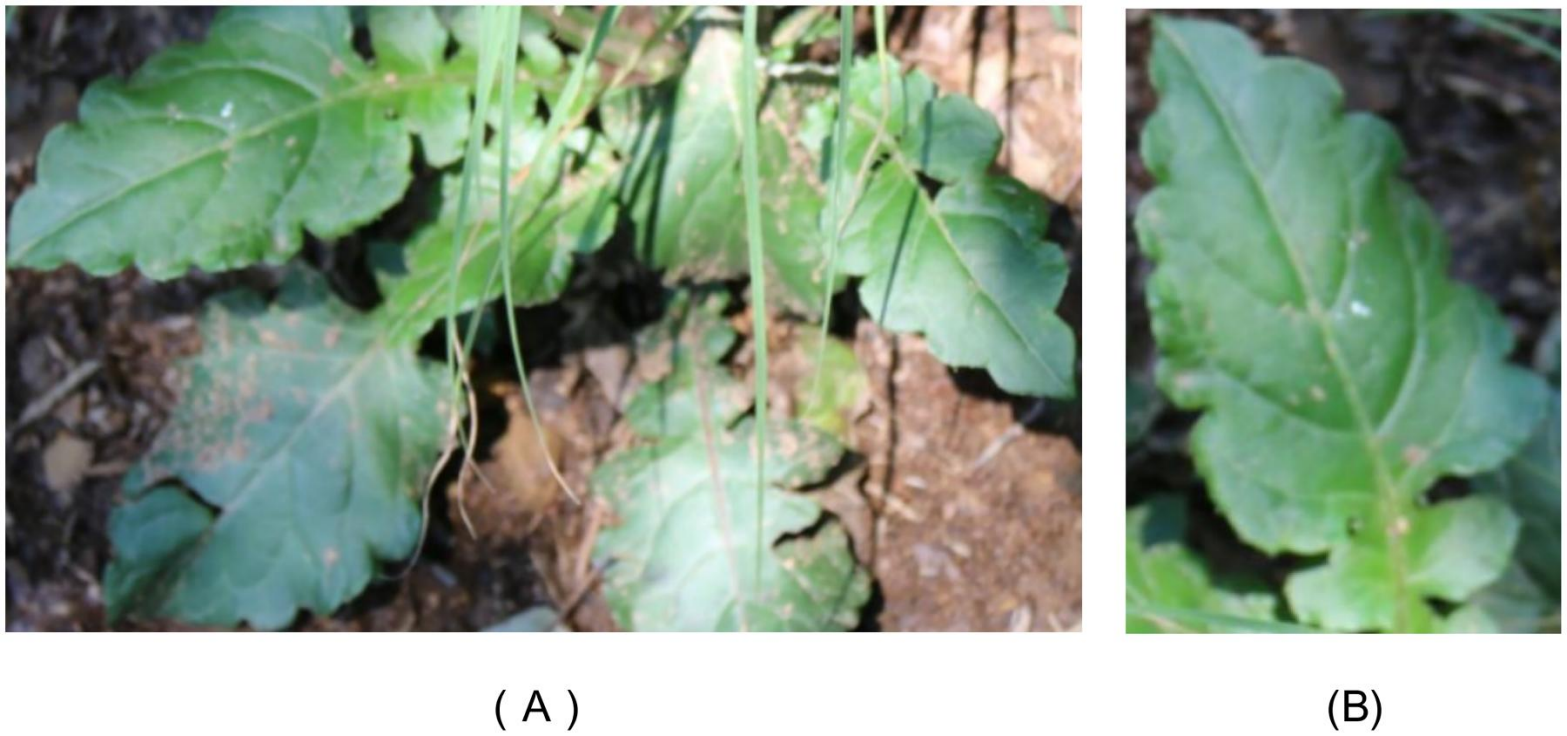
Leibnitzia anandria (linnaeus) turczaninow. The main performance of *leibnitzia anandria* is that the leaves are basal, larger, lotus-like, and are oblong-lanceolate in shape. (A): the photo of *leibnitzia anandria* was taken by the authors in Wanxian Mountain, Xinxiang City, Henan Province, China(coordinates: 113.6E, 35.7N);(B): the leaf of *leibnitzia anandria* from (A).

Samples of Leibnitzia anandria were collected from Wanxian Mountain, Xinxiang City, Henan Province, Chnia (113.6°E, 35.7°N). Total genomic DNA extracted from fresh leaf tissue using the Hipure SF Plant DNA Mini Kit following the manufacturer’s instruction (Magen). The specimen was deposited at Shanghai Origingene Biotechnology CO., Ltd. in Shanghai Zizhu International Education Park by author (binghua.ru@origin-gene.com) under voucher Number YS001489 (Figure 1).

### Genome sequencing, assembly, and annotation

The chloroplast genome of Leibnitzia anandria was sequenced by the next-generation sequencing (GenoLab M, Genemind sequencing platform). The quality of the sequenceing raw data was evaluated by FastQC (Andrews 2013), and trimmed using the software Cutadapt (Martin 2011) to obtain high-quality clean reads for subsequent analysis. The clean reads were de novo assembled and the complete chloroplast genome was obtained using assembly software NOVOPlast (Nicolas et al. 2017). The assembled genome was annotated by the software PGA (Qu et al. 2019), and manual correction was performed for all annotation results. A circular map of the chloroplast genome was generated by the online tool OGDRAW (Greiner et al. 2019). Relative synonymous codon usage (RSCU) in protein coding sequences of Leibnitzia anandria was determined in CodonW. Simple sequence repeats (SSRs) of Leibnitzia anandria was determined by MISA.

### Phylogenetic tree construction

To determine the phylogenetic relationship of Leibnitzia anandria, we used two datasets: the whole cp genome sequences from 14 related species of family Asteraceae, and the chloroplast DNA trnL-rpl32 sequences from these 14 spcies, along with 6 other chloroplast DNA trnL-rpl32 sequences from genus Leibnitzia and Gerbera, respectively. We also selected Codonopsis minima and Echinocodon lobophyllus from the family Campanulaceae as outgroups. The sequences of these species were downloaded from NCBI (http://www.ncbi.nlm.nih.gov/). All sequences were aligned using MAFFT software with default alignment parameters and then edited manually (Katoh et al. 2019). Conserved sequences among different species were identified using Gblock software. A maximum likelihood tree with bootstrap support was built using the RAxML software based on the GTRCATI model. The phylogenetic tree was visualized by software Itol version V1.4.4 (https://iTOL.embl.de/) (Letunic and Bork 2016).

## Result

**Figure 2.**
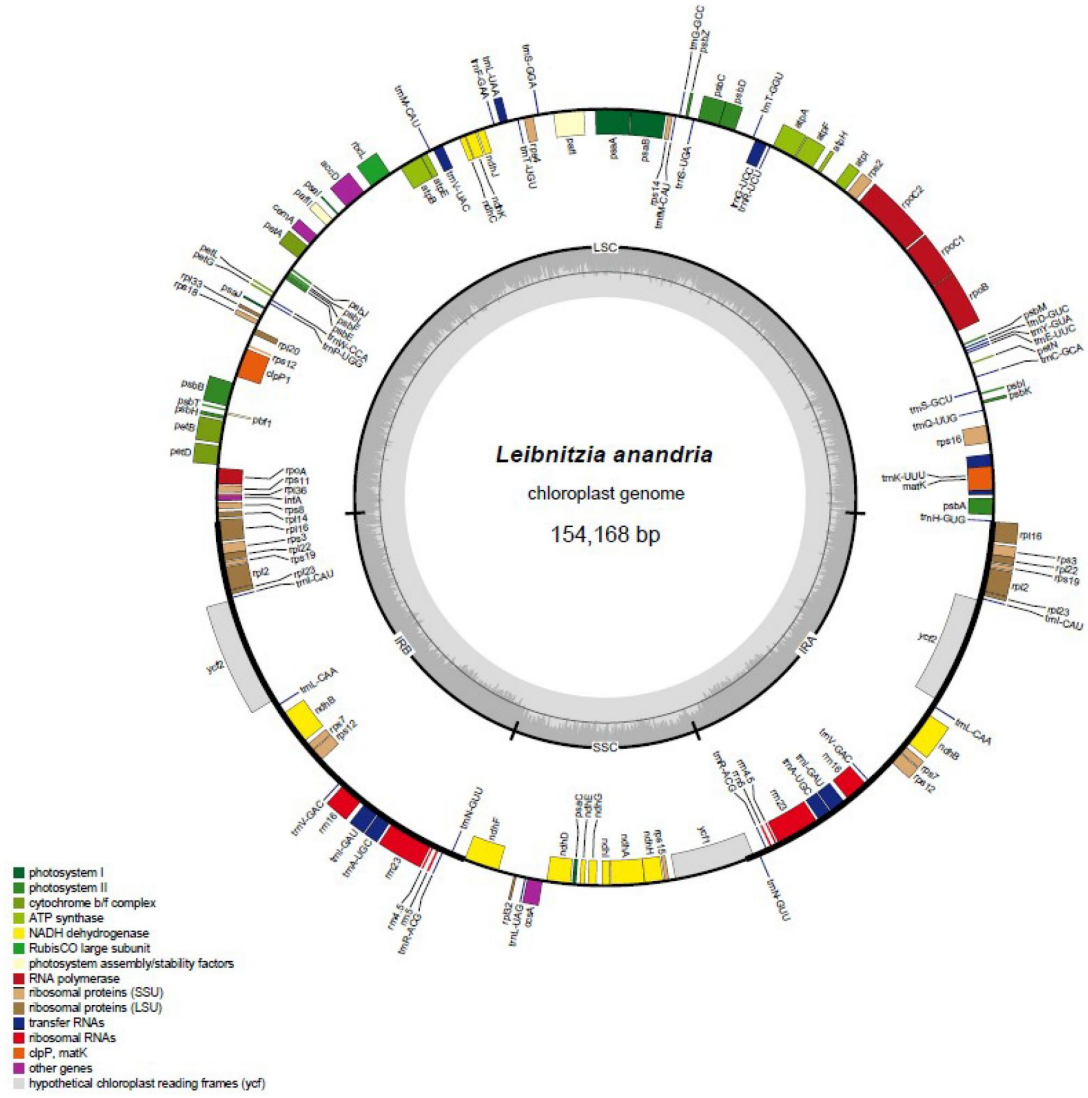
Genomic map of the leibnitzia anandria chloroplast genome generated by the online tool OGDRAW. Genes outside the circle are transcribed in a counterclockwise direction and those inside in a clockwise direction. LSC: large single-copy; SSC: small single-copy; IR: inverted repeat. The inner circle’s dashed region represents the GC content of the chloroplast genome of L. anandria. Genes belonging to different functional groups are represented using different colors.

By using next-generation high-throughput sequencing, 49455796 raw reads were obtained, which were filtered to yield 49448954 high-quality reads, with a total length of 7352487736 bp. The chloroplast genome of Leibnitzia anandria was assembled, with a total length of 154168 bp, GC content of 37.66%, and structure that includes a large single-copy region (LSC, 80166 bp), a small single-copy region (SSC, 18202 bp), and a pair of inverted repeat regions (IR, 27900 bp). A total of 133 genes were annotated, including 88 protein-coding genes, 37 tRNA-coding genes, and 8 rRNA-coding genes. Additionally, 26 simple sequence repeat(SSR) sequences were identified in the chloroplast genome of Leibnitzia anandria, among which mononucleotide repeats were the most abundant(12T, 10 A, and 1 G), followed by dinucleotide repeats (2AT), and one trinucleotide repeat (TCC) (Figure 2).

**Figure 3.**
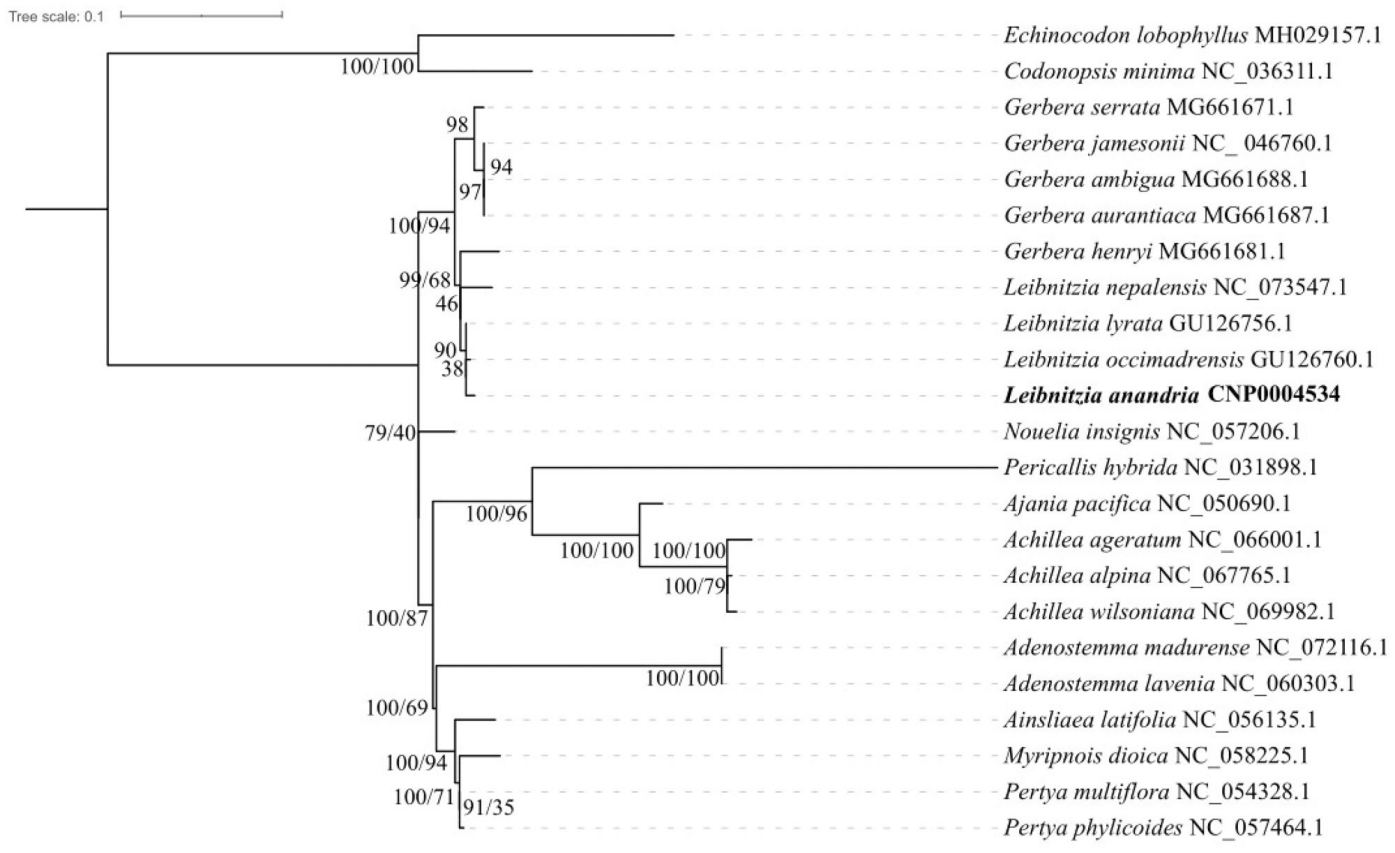
Maximum likelihood phylogenetic tree of leibnitzia anandria, and other 22 asteraceae species constructed using two different datasets. Bootstrap support values are given at the nodes, single bootstrap value form chloroplast DNA trnL-rpl32 sequences, two bootstrap value from left to right, respectively represented from the complete cp genome and from chloroplast DNA trnL-rpl32 sequences. Codonopsis minima and echinocodon lobophyllus were used as outgroups. The accession number of sequence of each plant species is shown after the species names:NC_046760.1(Zhang et al. 2019), NC_058225.1(Chen et al. 2019), NC_057206.1(Tian and Fu 2020), NC_057464.1(Wang et al. 2020), NC_054328.1(Jiang 2020), NC_067765.1(Niu 2021), NC_066001.1(Leonardo et al. 2022), NC_062413.1(Pires Paula 2020), NC_069982.1(Luo and Fu 2022), NC_072116.1(Kim et al. 2023), NC_060303.1(Li 2020), NC_053927.1(Luo and Pan 2019), NC_056135.1(Yin 2020), NC_050690.1(Kim and Kim 2020), NC_031898.1(Wang et al. 2015), NC_073547.1(Chen 2023), GU126760.1(Baird et al. 2010), GU126756.1(Baird et al. 2010), MG661671.1(Xu et al. 2018),MG661681.1(Xu et al. 2018),MG661688.1(Xu et al. 2018),MG661687.1(Xu et al. 2018), NC_036311.1(Cheon et al. 2017), MH029157.1(Wang et al. 2018).

To further investigate the phylogenetic relationship of Leibnitzia anandria, we selected 14 chloroplast genome sequences and 6 chloroplast DNA trnL-rpl32 sequences from family Asteraceae. Codonopsis minima and Echinocodon lobophyllus were chosen as outgroups. The GTRCATI model was determined as the best-fit model. We using the MAFFT method to align the genome dataset and the chloroplast DNA trnL-rpl32 dataset with the relevant data of Leibnitzia anandria, respectively, and constructed phylogenetic trees. The results indicated that the topologies of both datasets trees using ML methods were highly consistent with each other. Therefore, the topological stuctures of consensus phylogenetic trees with two datasets were integrated here with 2 support values on the branch (Figure 3). Single bootstrap value from chloroplast DNA trnL-rpl32 sequences, two bootstrap value from left to right, respectively represented from the complete cp genome and from chloroplast DNA trnL-rpl32 sequences. Additional, The original trees with two datasets were also represented in the Figure S1 and Figure S2.

The phylogenetic trees based on two datasets indicate that Leibnitzia anandria, along with other Leibnitzia species and closely related Gerbera species, form a distinct branch. Species from the Ainsliaea, Myripnois, and Pertya genera, which belong to the Mutisiae tribe (Funk et al. 2016), cluster together as a separate clade. On the other hand, the Nouelia genus, also belonging to the Mutisiae tribe (Funk et al. 2016), forms a separate branch. The results based on the trnL-rpl32 sequence indicate that Leibnitzia anandria is more closely related to L. occimadrensis and L. lyrate than to L. nepalensis. Interestingly, species G. henryi and species within the Leibnitzia genus cluster together instead of with other species from the Gerbera genus.

## Conclusion and discussion

In this research, we assembled the chloroplast genome of Leibnitzia anandria for the first time, revealing a 154,168 bp sequence that encodes 133 genes, including both large and small single-copy areas, alongside two inverted repeat regions. Sequencing depth confirmed the assembly’s comprehensive accuracy. Phylogenetic analysis showed that L. anandria forms a distinct clade with L. nepalensis and related Gerbera species, diverging from other Mutisieae members. Given the scarcity of complete chloroplast genomes for Leibnitzia and Gerbera, we highlighted the trnL-rpl32 region as a potential DNA barcode for phylogenetic studies (Cui et al. 2020). This study contributes six trnL-rpl32 sequences from Leibnitzia and Gerbera, compared against 15 other species’ sequences, including L. anandria’s. The results support the chloroplast genome findings and suggest a closer relationship between L. anandria and L. lyrate as well as L. occimadrensis than with L. nepalensis, confirming previous studies (Baird et al. 2010). Interestingly, G. henryi from the Gerbera genus appears more closely related to Leibnitzia than to other Gerbera species, indicating a particular link between Asian Gerbera and Leibnitzia species, in contrast to African Gerbera species (Xu et al. 2018). This study advances our understanding of L. anandria’s phylogeny, aiding in its taxonomic and evolutionary delineation.

## Supplementary Material

Supplemental Material

Supplemental Material

Supplemental Material

## Data Availability

The genome sequence data that supported the findings of this study are openly available in GeneBank of NCBI at https://www.ncbi.nlm.nih.gov under the accession No.PP566209. The associated BioProject, SRA, and Bio-Sample numbers are PRJNA1081144, SRR28110553 and SAMN40149392 respectively.
