# Supplementary figures and images for: The complete chloroplast genome sequence of leibnitzia anandria (linnaeus) turczaninow
Source: Mitochondrial DNA B Resour. 2024 May 2;9(5):578–82. doi: 10.1080/23802359.2024.2347511 (PMC11067557; doi:10.1080/23802359.2024.2347511)

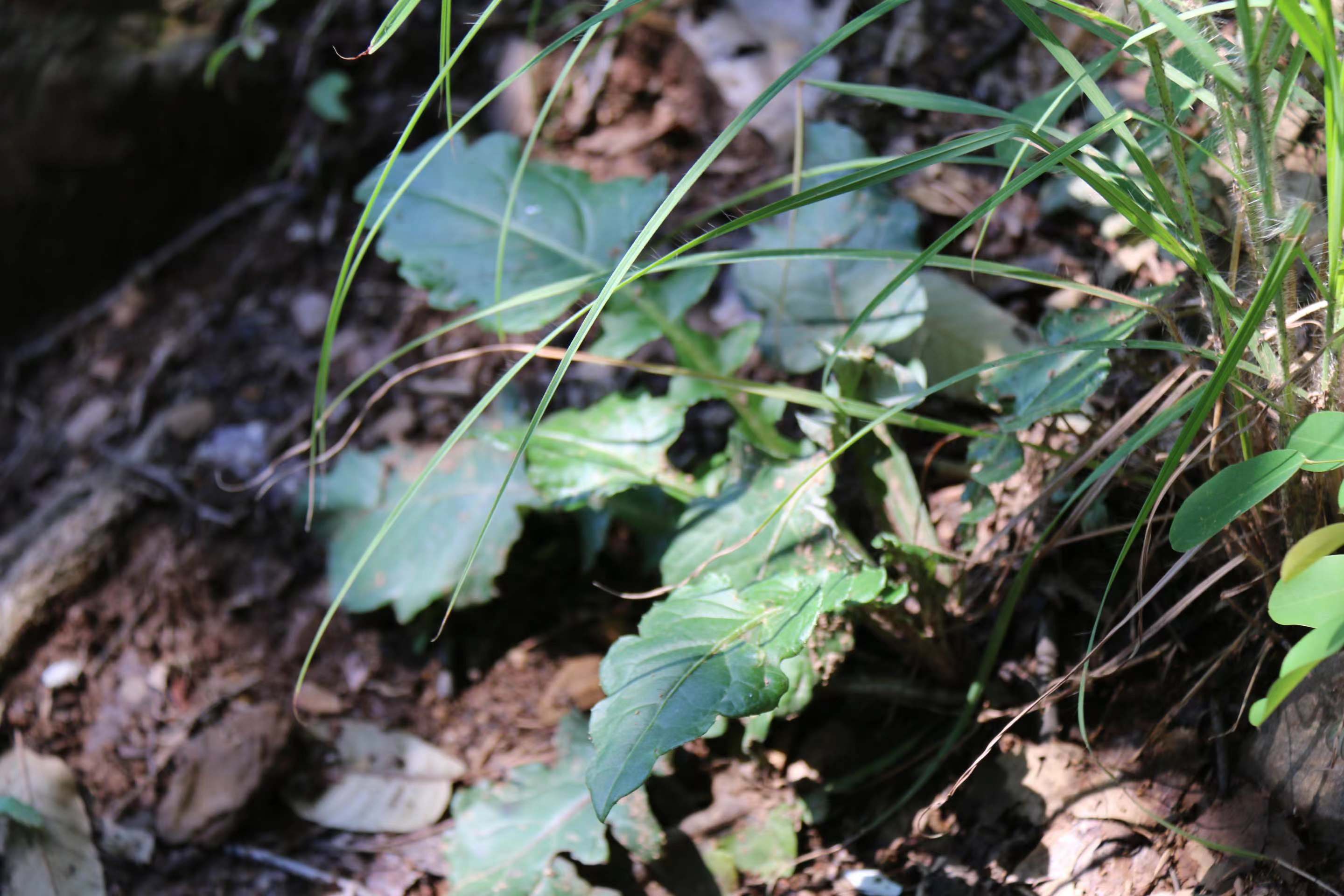

Supplement: Supplemental Material [file TMDN_A_2347511_SM0994.jpg]

Tree scale: 0.1

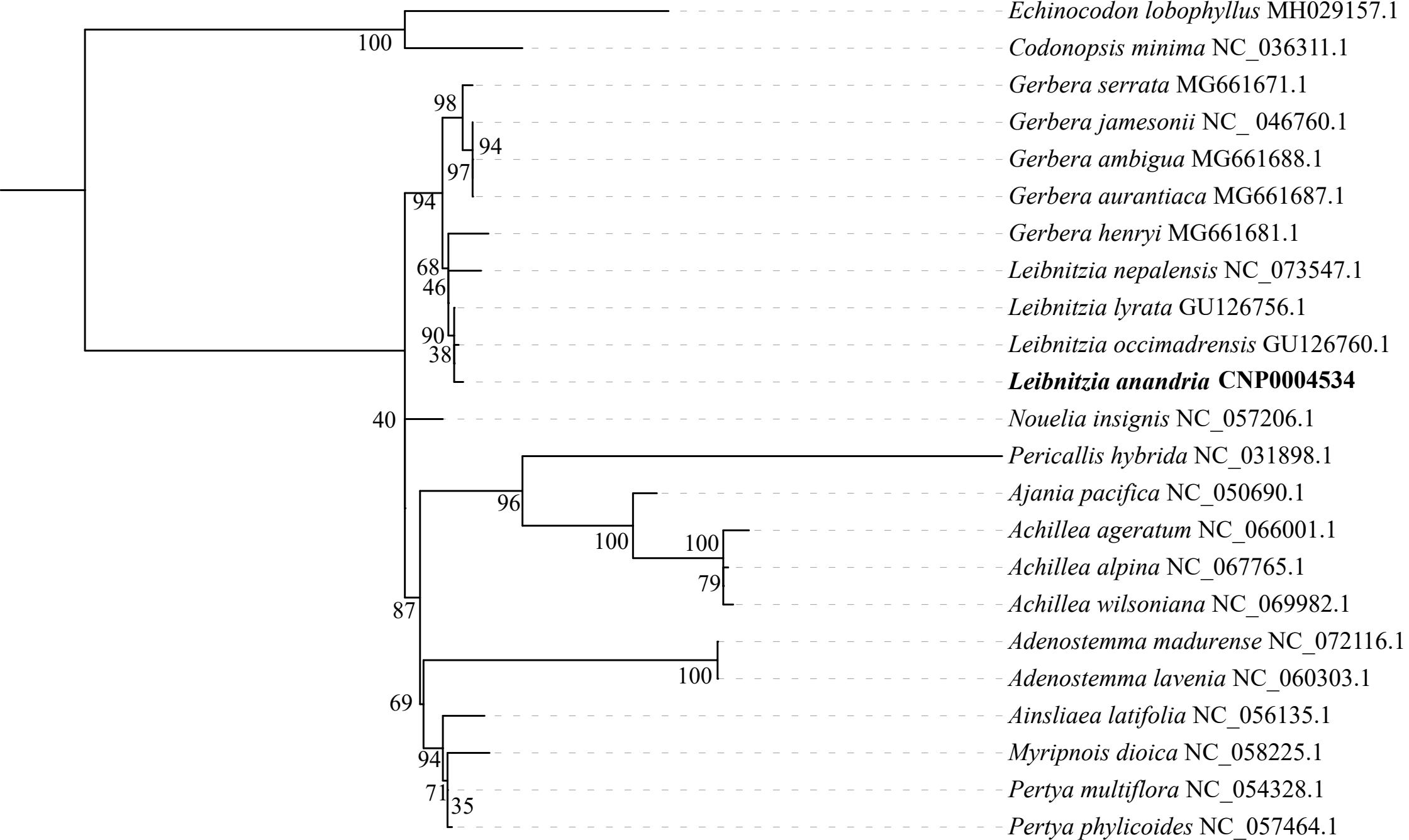

Supplement: Supplemental Material [file TMDN_A_2347511_SM0992.pdf]

Tree scale: 0.01

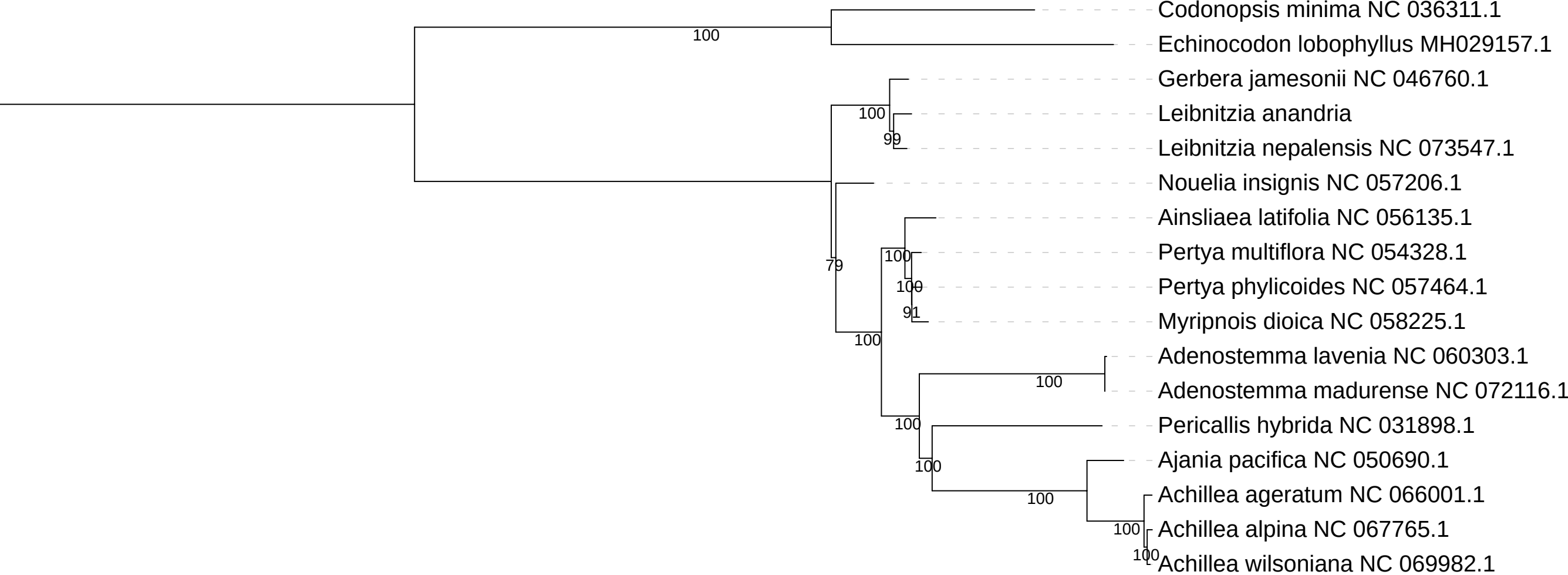

Supplement: Supplemental Material [file TMDN_A_2347511_SM0914.pdf]
